# A “patient as educator” intervention: Reducing stigmatizing attitudes toward mental illness among medical students

**DOI:** 10.3389/fpubh.2022.1020929

**Published:** 2022-12-21

**Authors:** Beatriz Atienza-Carbonell, Helena Hernández-Évole, Vicent Balanzá-Martínez

**Affiliations:** ^1^Department of Medicine, University of Valencia, Valencia, Spain; ^2^Faculty of Health Sciences, Valencian International University, Valencia, Spain; ^3^Teaching Unit of Psychiatry and Psychological Medicine, Department of Medicine, Universitat de València, Valencia, Spain; ^4^Center for Biomedical Research in Mental Health Network (CIBERSAM), Health Institute, Carlos III, Madrid, Spain; ^5^Evaluation Unit in Personal Autonomy, Dependency and Serious Mental Disorders (TMAP), University of Valencia, Valencia, Spain

**Keywords:** medical education, patient as educator, stigma, intervention, medical students

## Abstract

**Introduction:**

This pre-post quasi-experimental pilot study aimed to assess the degree of stigma toward mental illness and whether a single, direct-contact “patient as educator” intervention with people with mental illness can reduce the degree of stigma among medical students.

**Methods:**

All second-year medical students from the University of Valencia were invited to voluntarily complete the Community Attitudes Toward the Mentally Ill (CAMI), Reported and Intended Behavior Scale (RIBS), and Mental Health Knowledge Scale (MAKS) questionnaires before and after participating in the formal medical psychology course. A “patient as educator” workshop with expert patients was organized in the middle of the semester. A total of 127 students completed the survey; 20 students participated in the workshop (workshop group), and the remaining 107 students only took the formal educational course, forming the control group.

**Results:**

At baseline, the groups were demographically matched and did not differ in the components of stigma or knowledge of mental illness. After the intervention, a greater reduction in the CAMI subscales of authoritarianism and social restriction was observed in the workshop group than in the control group. In the workshop group, scores for the benevolence subscale of the CAMI decreased more among women than men. In the control group, scores for the authoritarianism and benevolence subscales of the CAMI increased and decreased significantly more, respectively, in women than men. No significant changes were observed in scores for the RIBS at post-intervention in either group.

**Discussion:**

The results of this pilot study suggest that a brief, direct-contact intervention in addition to formal medical education may further help reduce stigmatizing attitudes during the first years of medical school.

## 1. Introduction

In the social sciences field, social stigma was first defined as “the situation of an individual who is disqualified from full social acceptance” (1). From a social perspective, stigma refers to the adoption of discriminatory behaviors, prejudicial attitudes, negative emotional responses, and biased social structures toward members of a subgroup of society (2). In the general population, stigma related to mental illness or mental health services has been shown to be directly related to decreased active help-seeking behaviors for mental problems (3). Moreover, it has been observed that men present significantly higher levels of stigmatizing attitudes toward mental illness than women (4, 5).

For a significant proportion of the general population, the presence of rejection attitudes is more limiting and disabling for daily life than the mental illness itself (6). Furthermore, for people with mental illness, self-stigma represents a significant barrier as it leads to rejection, discrimination, and exclusion from social participation (7). Self-stigma is particularly detrimental when patients face negative attitudes from healthcare professionals. Although recent studies have suggested that negative attitudes toward people suffering from mental illness have improved in the general population, such attitudes remain prevalent among physicians (8, 9).

Most observational studies on attitudes and stigma toward mental illness have focused on the general population and healthcare workers, while only a few (10–13) have been conducted with medical students. Nonetheless, there is no evidence supporting the absence of stigma in this group. In fact, a recent systematic review of 128 studies from 20 countries shows that the prevalence of stigma against mental illness among medical students was as high as 97% (11). Moreover, recent studies have found much higher levels of stigma among medical students than other professionals in training (14). In addition, negative views about certain mental illnesses have been shown to be already present in the first 3 years of medical education, suggesting that stigma develops early during the preclinical period (7). However, Dilsad and Fidanoglu (15) observed that stigmatizing attitudes improved from the beginning to the end of the degree as a result of direct contact with people with mental illness; they also suggest that negative attitudes toward mental illness would be reduced during medical residency (15).

Growing scientific evidence suggests that participating in clinical rotations in psychiatric services and anti-stigma educational activities are two effective strategies to combat stigma toward mental illness among medical students (16–18). Moreover, proposed interventions to decrease stigma include, but are not limited to, direct contact with patients and indirect contact through educational films, lectures, role-playing, and education (19, 20). Overall, anti-stigma interventions for medical students should adopt a multi-dimensional approach that goes beyond theoretical knowledge and addresses, separately, the improvement of behaviors and attitudes toward those with mental health problems (7). The content of these interventions should also differ from those of campaigns targeting the general population (21).

According to a recent meta-analysis (22), only seven university-based studies on anti-stigma interventions have compared the efficacy of contact with people with mental illness (intervention group) with participation in an educational program (control group). However, in three of these seven studies, the anti-stigma contact intervention was indirect, including watching films (23, 24) and reading narratives (25). Of note, in the only study conducted with medical students (26), a single session of role-playing exercises did not significantly improve the students' stigmatizing attitudes. One other study found no differences between direct-contact and educational interventions (27).

In another recent meta-analysis of 90 studies (28), the immediate effects of anti-stigma interventions were measured; still, only two of them were conducted with college students, none of which encompassed medical students, and both were based on direct contact with people with mental illness (29, 30).

According to the direct-contact hypothesis, increased personal and professional interactions with people with mental illness are associated with more positive attitudes and less stigmatization toward them (10). Although patients have been shown to play a crucial role in medical education, their involvement tends to be passive, for example, through opportunistic patient contact in clinics and wards (31).

Several studies have suggested that students taught by trained patients acquire the same levels of competence in some medical areas (e.g., physical examination) as students taught by consultant physicians (32, 33). Nonetheless, the experience of participating in a lecture taught by a patient can increase the students' confidence, reduce anxiety, generate new insights, and change attitudes toward patients (33). Moreover, the involvement of patient educators in educational activities is not only beneficial for students but also has the potential to be valuable for patients at the personal level, both socially and therapeutically (34).

Despite several studies having addressed the importance of patients in medical education (31, 34), no recently published articles on anti-stigma have comprehensively discussed the crucial role of patients in decreasing negative attitudes toward people with mental illness among medical students. Therefore, this pilot study aimed: (i) to investigate whether medical students attending a course in medical psychology reduce the stigma attached to mental illness; (ii) to compare the effect of 'patient as educator' intervention on medical students in the workshop and control groups.

A significant improvement in stigma was expected in both groups. Further, the additional participation in a direct-contact intervention was expected to be associated with a greater reduction in stigmatizing attitudes compared with participating only in a course in Medical Psychology.

## 2. Materials and methods

### 2.1. Study design

This research is part of the VALencia Stigma in Medical Education (VALSME) research project, which has been evaluating the presence of stigma and mental health issues among medical students at the University of Valencia, Spain, since 2017 (35, 36). The study started before participants had contact with any theoretical content or academic activity specifically related to mental health and psychiatry. At our university, the first contact with these topics takes place during the medical psychology course, which takes place in the second year of the undergraduate medical curriculum.

This quasi-experimental pilot study followed a pre-post design. At the beginning of the medical psychology course (baseline), all second-year students were invited to complete an online survey, which included the Community Attitudes Toward the Mentally Ill (CAMI), Reported and Intended Behavior Scale (RIBS), and Mental Health Knowledge Scale (MAKS) questionnaires. Information on demographic variables was also collected, including sex, age, religion, and parental education level.

In the middle of the 4-month medical psychology course, a direct-contact “patient as educator” intervention was organized. It comprised a workshop led by three people with a mental illness diagnosis (i.e., experts by experience): one with schizophrenia and two with depression. Two of them were volunteers from ASIEM, a non-profit mental health association in Valencia, Spain; the third expert was a classmate of participants who suffered from depression and volunteered to participate in the intervention. These patient-educators shared their personal stories surrounding mental illness, including their experiences with symptoms, the impact that the diagnosis had on their lives, and how they dealt with the related stigma on a daily basis. Students participated voluntarily in this activity. The structure of the intervention, which can be considered as a workshop, was as follows: a 1-h lecture by patient-educators followed by a 90-min discussion with participants.

At the end of the course, all students were invited to complete the same survey conducted at baseline again. Students were asked to use their personal identity codes (detailed below) to allow matching the results of both surveys for each participant. The students who participated in the workshop and the course comprised the workshop group (WG), and those who only participated in the course comprised the control group (CG).

### 2.2. Selection criteria

**Figure 1:**
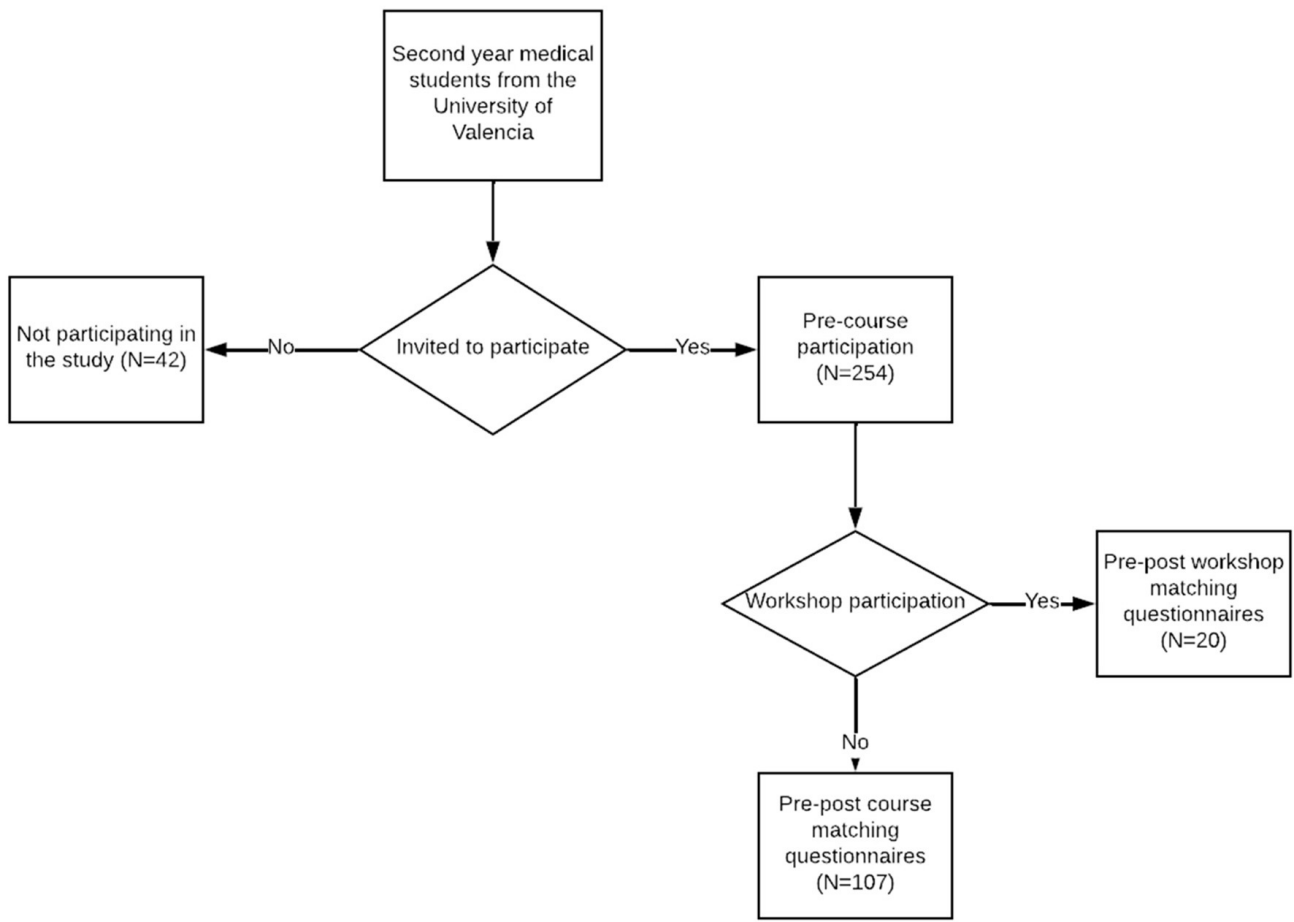
Study flow diagram.

The inclusion criteria for the VALSME pilot study were as follows: second-year medical students, no prior exposure to formal education in psychiatry/medical psychology, and voluntarily agreeing to participate in both evaluations (Figure 1). The exclusion criterion was failure to complete both questionnaires. Participants did not receive any financial or academic compensation for participation.

### 2.3. Instruments

Three self-administered questionnaires were used to assess different aspects of stigma toward mental illness.

#### 2.3.1. Community attitudes toward the mentally ill

The CAMI (37) assesses the different spatial variations in a population's response to mental health facilities. It comprises four subscales: authoritarianism, which evaluates opinions about people with mental illness being of a lower class than healthy individuals; benevolence, which evaluates acceptance attitudes toward patients, albeit this may represent a paternalistic attitude; social restriction, which assesses the danger that people with mental illness pose to society; community mental health ideology, which evaluates attitudes and beliefs related to the inclusion of people with mental illness in the community and society in general. This 40-item questionnaire has been validated in Spanish (38) and is rated on a 5-point Likert scale (1 = *strongly disagree*; 5 = *strongly agree*). Each subscale contains 10 statements regarding opinions on how to treat and care for people with severe mental illness, of which five are expressed as positive and the other five as negative. Higher scores on authoritarian and social restrictiveness imply more stigma, while higher scores on benevolence and community mental health ideology imply a higher acceptance of the mentally ill. Overall stigma was computed by summing up the subscales. Higher scores indicated more stigma against people with mental illness.

#### 2.3.2. Reported and intended behavior scale

The 8-item RIBS (39) assesses the presence of reported and future intentionality related to stigma toward mental illness. The first four items explore the prevalence of behaviors and they are not scored. Items 5–8 were scored on a 5-point Likert scale, with five answer options ranging from 1= strongly disagree and 5 = strongly agree. Overall score ranges from 0 to 20. Higher scores indicated more favorable expected behaviors.

#### 2.3.3. Mental health knowledge scale

The 12-item MAKS (40) assesses knowledge regarding mental illness stigma. MAKS comprises 6 stigma-related mental health knowledge areas: help seeking, recognition, support, employment, treatment, and recovery, and 6 items that inquire about knowledge of mental illness conditions. The items are scored on a 5-point Likert scale, ranging from 1 = strongly disagree and 5 = strongly agree. Total scores are calculated by adding together the response values of each item (“Don't know” was coded as neutral = 3). Items 6, 8, and 12 were reverse coded. Overall higher score indicated greater knowledge.

### 2.4. Statistical analysis

Quantitative variables were expressed as medians and interquartile ranges, with absolute numbers and frequencies being used to express categorical variables. Variable normality was assessed using Kolmogorov-Smirnov (CG) or Shapiro-Wilk tests (WG). To compare independent quantitative variables, Mann-Whitney's *U* test and Student's *t*-test were used. Pairwise comparisons were performed using Wilcoxon signed-rank test and paired sample *T*-tests. Effect sizes were calculated using *r* and Cohen's *d* depending on the statistical tests used. Chi-square and Fisher's exact tests were used to compare categorical variables given the reduced size of the WG. Statistical analysis was performed using SPSS software (version 24.0; IBM Corporation). Statistical significance was set at a *p* < 0.05.

### 2.5. Ethical considerations

Students were invited to complete a self-administered, confidential survey, and were informed that by agreeing to submit the questionnaires, they would be providing their informed consent to participate in the study. Participation was voluntary, and students could withdraw from the survey at any point before submitting the questionnaire. To maintain confidentiality and to contrast pre- and post-assessments, a personal identification code was generated for each participant based on the combination of the first letters of their given name, their father's name, their mother's name, and their day and month of birth (e.g., BEC2312). Anonymity of the collected data was guaranteed by participation. Data were saved in an offline database for statistical analysis.

This study involved human participants but was not approved by an ethics committee. According to the Ethics Committee of the University of Valencia, ethical approval was waived because the study did not include any medical or biomedical intervention. Participants provided informed consent to participate in the study before participating.

## 3. Results

### 3.1. Sample description

In total, data from 127 second-year students were analyzed (out of 296; response rate: 42.9%). Descriptions of the main sociodemographic variables of the sample are shown in Table 1. Most participants were women (76.4%) and between the ages of 18–20 years (96.1%).

**Table 1:** Sociodemographic characteristics of the sample at baseline by group.

	**Variable**	**Control group (*n* = 107) *N* (%)**	**Workshop group (*n* = 20) *N* (%)**	***F***	***p***
Age	18–20	102 (95.3)	20 (100.0)	0.973^a^	0.594
	21–23	5 (4.7)	0 (0.0)		
Gender	Men	27 (25.2)	3 (15.0)	0.978^a^	0.402
	Women	80 (74.8)	17 (85.0)		
Religion	Atheist or agnostic	68 (63.6)	12 (60.0)	4.638	0.400
	Christian	36 (33.6)	7 (35.0)		
	Other	3 (2.8)	1 (5.0)		
Father's level of education	Primary	29 (27.1)	5 (25.0)	1.081	0.644
	Medium	13 (12.1)	4 (20.0)		
	University	65 (60.7)	11 (55.0)		
Mother's level of education	Primary	21 (19.6)	3 (15.0)	1.353^a^	0.489
	Medium	16 (15.0)	5 (25.0)		
	University	70 (65.4)	12 (60.0)		

^a^Pearson Chi-square test.

Twenty students (85% women) participated in the WG and 107 in the CG. At the beginning of the study, the sociodemographic characteristics of both groups were similar (Table 1).

### 3.2. Baseline stigma

For the total sample (*N* = 127), more than half had had contact with people with mental illness, either as a cohabitant (39.4%), neighbor (38.6%), or friend (50.4%). However, only 14.9% had worked with people with mental illness. Stigma levels did not differ by sex. At study onset, no significant differences were found by group regarding the total scores for the CAMI, RIBS, and MAKS, nor in the four subscales of the CAMI (in all cases, *p* > 0.05) and in the reported behavior items of the RIBS (Table 2).

**Table 2:** Results for the reported and intended behavior scale—reported behavior items at study onset by group.

**Reported and intended behavior scale—reported behavior items**	**Control group (*n* = 107) *N* (%)**	**Workshop group (*n* = 20) *N* (%)**	***F***	***p***
Are you currently living with, or have you ever lived with, someone with a mental health problem?	43 (30.2)	7 (35.0)	1.219	0.614
Are you currently working with, or have you ever worked with, someone with a mental health problem?	15 (14.0)	4 (20.0)	0.657	0.729
Do you currently have, or have you ever had, a neighbor with a mental health problem?	37 (34.6)	12 (60.0)	4.170	0.118
Do you currently have, or have you ever had, a close friend with a mental health problem?	58 (54.2)	6 (30.0)	4.345	0.098

### 3.3. Within-group comparison of changes in stigma

In the WG, the total scores for the CAMI (*p* = 0.014; *d* = 0.65) and its authoritarianism subscale (*p* = 0.004, *r* = 0.46) decreased significantly at post-intervention with a large and medium size effect, respectively. Meanwhile, the score for the social restriction subscale of the CAMI significantly increased (*p* = 0.003; *r* = 0.47) at post-intervention with a medium size effect. Regarding MAKS knowledge of mental illness conditions items, a significant decrease in the perception of stress regarding mental illness was observed at post-intervention (*p* = 0.022). However, no significant differences between pre- and post-intervention scores were found in the other subscales of the CAMI and the total score for the RIBS (see Table 3).

**Table 3:** Comparison of pre- and post-scores for the community attitudes toward the mentally ill, reported and intended behavior scale, and mental health knowledge scale in both groups.

	**Variable**	**Pre-intervention control group (*n* = 107) median (IQR)**	**Post-intervention control group (*n* = 107) median (IQR)**	**Z**	***p***	***r***	**Pre-intervention workshop group (*n* = 20) median (IQR)**	**Post-intervention workshop group (*n* = 20) median (IQR)**	**Z**	***p***	***r***
CAMI	Authoritarianism	39 (36, 41)	40 (36, 43)	−2.401	0.016	0.16	38.5 (33.5, 40)	41 (37, 43.75)	−2.902	0.004	0.46
	Benevolence	16 (14, 20)	15 (13, 18)	−1.790	0.073	0.12	17 (14, 19)	16 (12, 18.75)	−1.236	0.216	0.20
	Social restriction	42 (39, 45)	43 (40, 46)	−2.222	0.026	0.15	41 (38, 43)	44.5 (41, 47)	−2.971	0.003	0.47
	CMHI*	19.61 ± 5.10	19.36 ± 7.05	0.409	0.683	0.04	21.2 ± 4.56	19.35 ± 5.18	1.834	0.082	0.41
	CAMI Total score*	116.64 ± 4.93	117.33 ± 5.95	−1.057	0.293	0.10	115.45 ± 5.680	119 ± 5.2	−2.911	0.009	0.65
MAKS total score		24 (22, 26)	25 (24, 26)	−4.481	< 0.001	0.31	23 (21.25, 24)	25 (24, 27)	−2.623	0.009	0.41
RIBS total score		16 (15, 19)	17 (15, 20)	−1.758	0.079	0.12	17 (14, 19)	18 (13.5, 19)	−0.693	0.488	0.11

^*^For these variables, paired sample T-test (mean ± standard deviation; Cohen's d for effect size) was used. CAMI, community attitudes toward the mentally ill; CMHI, community mental health ideology subscale; MAKS, mental health knowledge scale; RIBS, reported and intended behavior scale.

In the CG, no significant differences between pre- and post-intervention were observed in the total scores for the CAMI and the RIBS. Conversely, the scores for the authoritarianism subscale of the CAMI (*p* = 0.016; *r* = 0.16) decreased significantly at post-intervention with a small size effect. Additionally, the scores for the social restriction subscale of the CAMI decreased significantly at post-intervention (*p* = 0.026; *r* = 0.15; Table 3) with a small size effect. Regarding the MAKS knowledge of mental illness conditions items, the acknowledgment of depression as a mental illness worsened from pre- to post-intervention (*p* = 0.038), and no changes were observed in the scores about other mental illnesses.

### 3.4. Between-group comparison of changes in stigma

The reduction in the scores for the authoritarianism (*p* = 0.006) and social restriction subscales of the CAMI (*p* = 0.015) from pre- to post-intervention was significantly higher in the WG group than in the CG, with medium effect sizes (*r* = 0.31 and *r* = 0.30, respectively). No differences were observed in the remaining stigma variables (Table 4).

**Table 4:** Between-group comparison for changes in stigma.

	**Variable**	**Control group (*n* = 107) median (IQR)**	**Workshop group (*n* = 20) median (IQR)**	***U***	***p***	***r***
CAMI	Authoritarianism	1 (−1, 3)	4 (1.5, 5)	659.5	0.006	0.31
	Benevolence	0 (−3, 2)	−2 (−4, 2)	943.5	0.400	0.07
	Social restriction	1 (−1, 4)	3 (1.5, 4.75)	703.5	0.015	0.30
	CMHI*	−0.252 ± 6.384	−1.85 ± 4.510	1.069	0.287	0.08
	Total score*	0.682 ± 6.678	3.55 ± 5.453	−1.809	0.073	0.17
MAKS total score		1 (−2, 5)	2 (−2, 3)	1056.5	0.929	0.01
RIBS total score		0 (0, 2)	0.5 (−1.75, 2)	1053	0.909	0.01

*For these variables, Student's t-test (Mean ± standard deviation; Cohen's d for effect size) was used. CAMI, community attitudes toward the mentally ill; CMHI, community mental health ideology subscale; MAKS, mental health knowledge scale; RIBS, reported and intended behavior scale.

### 3.5. Relationship of changes in stigma with sociodemographic variables

The Mann-Whitney *U* test was conducted to determine potential sex differences in changes of stigma variables. In the WG, scores for the benevolence subscale of the CAMI decreased more from pre- to post-intervention among women than men (*U* = 5.5; *p* = 0.033). In the CG, the increase in the scores for the authoritarianism subscale of the CAMI (*U* = 758; *p* = 0.020) and the decrease in the scores for the benevolence subscale of the CAMI were significantly higher in women than men (*U* = 795; *p* = 0.040). For both groups, no significant differences were found according to age, religion, parental education level, and contact with people with mental illness.

## 4. Discussion

This pilot, pre-post, quasi-experimental, pilot study aimed to examine whether a formal educational program can reduce the degree of stigma toward mental illness, and whether additional participation in a direct-contact “patient as educator” intervention could significantly further reduce stigmatizing attitudes (vs. participation in the formal educational program alone) among medical students at the University of Valencia. This research is among the few experimental studies examining the effects of direct-contact interventions on stigmatizing attitudes among medical students (15, 26).

In this study, a formal educational program on medical psychology increased the students' overall knowledge about mental health conditions. Nevertheless, no significant reduction in stigma was observed when students were only exposed to the formal educational program. Our results are consistent with previous evidence suggesting that specific training in mental illness-related stigma may improve knowledge about mental health, but not the attitudinal and behavioral aspects of stigma toward mental health (41). It is of great concern that stigmatization and negative perceptions occur among medical students despite the provision of medical education and information about mental illnesses and their treatments (9, 12, 13). Therefore, anti-stigma interventions should adopt a multi-dimensional approach, go beyond theoretical knowledge, and separately target and assess improvements in attitudes and behaviors (7, 41).

As expected, our results suggest that additional participation in a direct-contact intervention with people with mental illness can significantly reduce stigmatizing attitudes among medical students. Nonetheless, such diminishment did not occur when students attended just the course in Medical Psychology. The concomitant use of direct-contact interventions and formal medical education may help improve the medical students' perceptions of people with mental illness as equals to the general population and reduce their perceptions about the dangerousness of this group.

As aforementioned, the evidence supporting the effectiveness of different modalities and approaches for reducing stigma toward people with mental illness in different healthcare professions is inconsistent (7). Although several studies conducted with university students have found that both direct-contact and specific educational interventions can reduce stigma compared with a control condition (27, 42–44), none were conducted among medical students. A previous study (45) found that a short-term, direct-contact intervention was effective in decreasing stigma in second-year social work students, concurring with the results of the present study. However, this cited research combined direct contact with other anti-stigma strategies, and stigma was assessed using an *ad hoc* instrument, not the validated questionnaires used in our study.

A growing body of literature has examined differences in stigma according to sex (37, 46), and the present findings further confirm that women show more humanitarian attitudes toward people with mental illness. Moreover, researchers have described an inverse relationship between contact with a person with mental illness and endorsing stigma and discrimination (47). Nevertheless, in our study, contact with people with mental illness did not affect changes in stigma levels after the direct-contact intervention.

### 4.1. Study limitations and strengths

The results of this study should be considered in the context of some methodological limitations. Importantly, the sample size was relatively small, especially that of the experimental group, as participation in the workshop was voluntary. Hence, the study might not have had sufficient power to detect significant differences between the groups, for example, a type II error. Moreover, despite the participation rate being higher than 40%, concerns regarding the representativeness of the sample cannot be entirely ruled out. The students were not randomized into the groups; however, quasi-experimental designs are usually necessary within the educational context, as was the case in this study. The students' responses could be subjected to social desirability bias, although the online nature of the survey and the anonymization of the responses are likely to have reduced this possibility.

Despite these limitations, we believe that this study is relevant for several reasons. First, the groups were well-matched at baseline, not differing in any of the stigma components nor in the knowledge of mental illness. Second, this is one of the few experimental studies to examine the effectiveness of a single, direct-contact “patient as educator” intervention with people with mental illness aimed to reduce stigma in medical students (15, 26). Third, although similar studies exist in the context of other health science students (30, 42, 48, 49), this is the first study conducted with medical students in Spain. Fourth, stigma was assessed using three complementary and validated questionnaires, allowing for a detailed and multidimensional assessment of stigmatizing attitudes toward mental illness.

## 5. Conclusions

Based on the present results, further research should elucidate whether the effect of a direct-contact intervention is sustained over time, as well as whether targeted interventions with a longer duration and administered periodically have an even greater effect than single-encounter interventions. Future studies with larger sample sizes are also warranted to better clarify the effects and sustainability of direct-contact interventions over time in future clinicians and other healthcare professionals. Fostering positive attitudes among medical students, who may be more willing to learn, may be easier than prompting healthcare professionals to reduce their established negative attitudes toward mental health. This, in turn, may lead to better care for people with mental illness by these future clinicians.

## Data availability statement

The raw data supporting the conclusions of this article will be made available by the authors, without undue reservation.

## Ethics statement

Ethical approval was waived by Ethics Committee of the University of Valencia because the study did not include any medical or biomedical intervention. Participants provided their written informed consent prior to participating in this study.

## Author contributions

Conceptualization, methodology, investigation, writing original draft preparation, and review and editing: BA-C, HH-É, and VB-M. Software and formal analysis: BA-C. Supervision: VB-M. All the authors have read and agreed to the current version of the manuscript.

## References

[1] Goffman E. Stigma and social identity. In:Goffman E, editor. Stigma: Notes on the Management of Spoiled Identity. London: Penguin (1963), p. 3.

[2] Corrigan PW. Mental health stigma as social attribution: implications for research methods and attitude change. Clin Psychol. (2000) 7:48–67. 10.1093/clipsy.7.1.48

[3] Schnyder N Panczak R Groth N Schultze-Lutter F. Association between mental health-related stigma and active help-seeking: systematic review and meta-analysis. Br J Psychiatry. (2017) 210:261–8. 10.1192/bjp.bp.116.189464 28153928

[4] Latalova K Kamaradova D Prasko J. Perspectives on perceived stigma and self-stigma in adult male patients with depression. Neuropsychiatr Dis Treat. (2014) 10:1399–405. 10.2147/NDT.S54081 25114531 PMC4122562

[5] Oliffe JL Ogrodniczuk JS Gordon SJ Creighton G Kelly MT Black N. Stigma in male depression and suicide: a Canadian sex comparison study. Commun Ment Health J. (2016) 52:302–10. 10.1007/s10597-015-9986-x 26733336 PMC4805721

[6] Pietrus M. Report stigma is more life-limiting and disabling than the illness itself. Can J Psychiatr. (2014) 59:S3. 10.1177/070674371405901S01 25565700 PMC4213750

[7] Suwalska J Suwalska A Neumann-Podczaska A Łojko D. Medical students and stigma of depression. Part I Stigmatization of patients. Psychiatr Pol. (2016) 51:495–502. 10.12740/PP/OnlineFirst/63515 28866719

[8] Mukherjee R Fialho A Wijetunge A Checinski K Surgenor T. The stigmatisation of psychiatric illness: the attitudes of medical students and doctors in a London teaching hospital. Psychiatr Bull. (2002) 26:178–81. 10.1192/pb.26.5.178

[9] Youssef FF. Attitudes toward mental illness among Caribbean medical students. Educ Health. (2018) 31:3–9. 10.4103/1357-6283.239029 30117466

[10] Chang S Ong HL Seow E Chua BY Abdin E Samari E. Stigma towards mental illness among medical and nursing students in Singapore: a cross-sectional study. BMJ Open. (2017) 7:e018099. 10.1136/bmjopen-2017-018099 29208617 PMC5719274

[11] Gervas R Bueno G Garcia-Ullan L de La Mata R Roncero C. Is there a stigma towards mental illness among medical students? A systematic review of the 1997–2018 Literature. J Evol Med Dent Sci. (2020) 9:299–304. 10.14260/jemds/2020/67

[12] Oliveira AM Machado D Fonseca JB Palha F Silva Moreira P Sousa N. Stigmatizing attitudes toward patients with psychiatric disorders among medical students and professionals. Front Psychiatry. (2020) 11:326. 10.3389/fpsyt.2020.00326 32425827 PMC7207477

[13] Babicki M Małecka M Kowalski K Bogudzińska B Piotrowski P. Stigma levels toward psychiatric patients among medical students: a worldwide online survey across 65 countries. Front Psychiatry. (2021) 12:909. 10.3389/fpsyt.2021.798909 34966314 PMC8710677

[14] Gonzales L. The role of right-wing authoritarianism in distinguishing mental health stigma among treatment providers. Stigma Health. (2022) 7:62–9. 10.1037/sah0000316

[15] Ay P Dilsad S Fidanoglu O. Does stigma concerning mental disorders differ through medical education? A survey among medical students in Istanbul. Soc Psychiatry Psychiatr Epidemiol. (2006) 41:63–7. 10.1007/s00127-005-0994-y 16328750

[16] Cutler JL Harding KJ Hutner LA Clarissa C Graham MJ. Reducing medical students' stigmatization of people with chronic mental illness: a field intervention at the “living museum” state hospital art studio. Acad Psychiatry. (2012) 36:191–6. 10.1176/appi.ap.10050081 22751820

[17] Lyons Z. Impact of the psychiatry clerkship on medical student attitudes towards psychiatry and to psychiatry as a career. Acad Psychiatry. (2014) 38:35–42. 10.1007/s40596-013-0017-3 24464416

[18] Mino Y Yasuda N Tsuda T Shimodera S. Effects of a one-hour educational program on medical students' attitudes to mental illness. Psychiatry Clin Neurosci. (2001) 55:501–7. 10.1046/j.1440-1819.2001.00896.x 11555346

[19] Stubbs A. Reducing mental illness stigma in health care students and professionals: a review of the literature. Aust Psychiatry. (2015) 22:579–84. 10.1177/1039856214556324 25371444

[20] Yamaguchi S Wu S-I Biswas M Yate M Aoki Y Barley EA. Effects of short-term interventions to reduce mental health–related stigma in university or college students: a systematic review. J Nerv Ment Dis. (2013) 201:490–503. 10.1097/NMD.0b013e31829480df 23719324

[21] Ungar T Knaak S. The hidden medical logic of mental health stigma. Aust N Z J Psychiatry. (2013) 47:611–2. 10.1177/0004867413476758 23405014

[22] Morgan AJ Reavley NJ Ross A Too LS Jorm AF. Interventions to reduce stigma towards people with severe mental illness: systematic review and meta-analysis. J Psychiatr Res. (2018) 103:120–33. 10.1016/j.jpsychires.2018.05.017 29843003

[23] Chan JYN Mak WWS Law LSC. Combining education and video-based contact to reduce stigma of mental illness: “The Same or Not the Same” anti-stigma program for secondary schools in Hong Kong. Soc Sci Med. (2009) 68:1521–6. 10.1016/j.socscimed.2009.02.016 19282079

[24] Corrigan PW Larson J Sells M Niessen N Watson AC. Will filmed presentations of education and contact diminish mental illness stigma? Commun Ment Health J. (2007) 43:171–81. 10.1007/s10597-006-9061-8 16988883

[25] Mann CE Himelein MJ. Putting the person back into psychopathology: an intervention to reduce mental illness stigma in the classroom. Soc Psychiatry Psychiatr Epidemiol. (2008) 43:545–51. 10.1007/s00127-008-0324-2 18286216

[26] Roberts LM Wiskin C Roalfe A. Effects of exposure to mental illness in role-play on undergraduate student attitudes. Fam Med. (2008) 40:477–83. 18928074

[27] Kosyluk K. Investigating the Impact of Education and Contact-Based Anti-Stigma Interventions on the Stigma of Mental Illness in the College Population. Illinois: Illinois Institute of Technology (2014).

[28] Maunder RD White FA. Intergroup contact and mental health stigma: a comparative effectiveness meta-analysis. Clin Psychol Rev. (2019) 72:101749. 10.1016/j.cpr.2019.101749 31254936

[29] Giacobbe MR Stukas AA Farhall J. The effects of imagined vs. actual contact with a person with a diagnosis of schizophrenia. Basic App Soc Psychol. (2013) 35:265–71. 10.1080/01973533.2013.785403

[30] Nguyen E Chen TF O'Reilly CL. Evaluating the impact of direct and indirect contact on the mental health stigma of pharmacy students. Soc Psychiatry Psychiatr Epidemiol. (2012) 47:1087–98. 10.1007/s00127-011-0413-5 21755345

[31] Howe A Anderson J. Involving patients in medical education. BMJ. (2003) 327:326–8. 10.1136/bmj.327.7410.326 12907488 PMC169640

[32] Hendry GD Schrieber L Bryce D. Patients teach students: partners in arthritis education. Med Educ. (1999) 33:674–7. 10.1046/j.1365-2923.1999.00524.x 10476018

[33] Wykurz G Kelly D. Developing the role of patients as teachers: literature review. BMJ. (2002) 325:818–21. 10.1136/bmj.325.7368.818 12376445 PMC128951

[34] Lauckner H Doucet S Wells S. Patients as educators: the challenges and benefits of sharing experiences with students. Med Educ. (2012) 46:992–1000. 10.1111/j.1365-2923.2012.04356.x 22989133

[35] Atienza-Carbonell B Balanzá-Martínez V. Prevalence of depressive symptoms and suicidal ideation among Spanish medical students. Actas Esp Psiquiatr. (2020) 48:154–62. 32920780

[36] Atienza-Carbonell B Guillén V Irigoyen-Otiñano M Balanzá-Martínez V. Screening of substance use and mental health problems among Spanish medical students: a multicenter study. J Affect Disord. (2022) 311:391–8. 10.1016/j.jad.2022.05.090 35609765

[37] Taylor SM Dear MJ. Scaling community attitudes toward the mentally ill. Schizophr Bull. (1981) 7:225–40. 10.1093/schbul/7.2.225 7280561

[38] Ochoa S Martínez-Zambrano F Vila-Badia R Arenas O Casas-Anguera E García-Morales E. Spanish validation of the social stigma scale: community attitudes towards mental illness. Rev Psiquiatr Salud Ment. (2016) 9:150–7. 10.1016/j.rpsmen.2015.02.002 25882535

[39] Evans-Lacko S Rose D Little K Flach C Rhydderch D Henderson C. Development and psychometric properties of the reported and intended behaviour scale (RIBS): a stigma-related behaviour measure. Epidemiol Psychiatr Sci. (2011) 20:263–71. 10.1017/S2045796011000308 21922969

[40] Evans-Lacko S Little K Meltzer H Rose D Rhydderch D Henderson C. Development and psychometric properties of the mental health knowledge schedule. Can J Psychiatry. (2010) 55:440–8. 10.1177/070674371005500707 20704771

[41] Kassam A Glozier N Leese M Loughran J Thornicroft G A. controlled trial of mental illness related stigma training for medical students. BMC Med Edu. (2011) 11:51. 10.1186/1472-6920-11-51 21801355 PMC3161004

[42] Clement S van Nieuwenhuizen A Kassam A Flach C Lazarus A de Castro M. Filmed vs. live social contact interventions to reduce stigma: randomised controlled trial. Br J Psychiatry. (2012) 201:57–64. 10.1192/bjp.bp.111.093120 22157800

[43] Corrigan PW River LP Lundin RK Penn DL Uphoff-Wasowski K Campion J. Three strategies for changing attributions about severe mental illness. Schizophr Bull. (2001) 27:187–95. 10.1093/oxfordjournals.schbul.a006865 11354586

[44] Matteo EK You D. Reducing mental illness stigma in the classroom. Teach Psychol. (2012) 39:121–4. 10.1177/0098628312437720

[45] Shera W Delva-Tauiliili J. Changing MSW students' attitudes towards the severely mentally ill. Commun Ment Health J. (1996) 32:159–69. 10.1007/BF02249753 8777872

[46] Pascucci M La Montagna M Di Sabatino D Stella E Nicastro R Grandinetti P. Stigma and attitudes towards mental illness: gender differences in a sample of Italian medical students. Eur Psychiatr. (2017) 41:S739–S739. 10.1016/j.eurpsy.2017.01.1359

[47] Corrigan PW Watson AC. Understanding the impact of stigma on people with mental illness. World Psychiatry. (2002) 1:16–20. 16946807 PMC1489832

[48] Martínez-Martínez C Sánchez-Martínez V Sales-Orts R Dinca A Richard-Martínez M Ramos-Pichardo JD. Effectiveness of direct contact intervention with people with mental illness to reduce stigma in nursing students. Int J Ment Health Nurs. (2019) 28:735–43. 10.1111/inm.12578 30693628

[49] Patten SB Remillard A Phillips L Modgill G Szeto AC Kassam A. Effectiveness of contact-based education for reducing mental illness-related stigma in pharmacy students. BMC Med Edu. (2012) 12:120. 10.1186/1472-6920-12-120 23216787 PMC3533989

